# Tanshinone IIA alleviates IL-1β-induced chondrocyte apoptosis and inflammation by regulating FBXO11 expression

**DOI:** 10.1016/j.clinsp.2024.100365

**Published:** 2024-04-26

**Authors:** Jin Xu, XiaoCheng Zhi, YunHui Zhang, Ren Ding

**Affiliations:** Department of Orthopaedics, Shanghai Baoshan Hospital of Integrated Traditional Chinese and Western Medicine, Shanghai City, China

**Keywords:** Chondrocytes, Il-1β, Tanshinone iia, Fbxo11, PI3K/AKT, NF-κB

## Abstract

**Objective:**

This study explored the pharmacological mechanism of Tanshinone IIA (TAN IIA) in the treatment of Osteoarthritis (OA), which provided a certain reference for further research and clinical application of Tan IIA in OA.

**Methods:**

CHON-001 cells were stimulated with 10 μg/mL IL-1β for 48 h and treated with 10 μM TAN IIA for 48 h. Cellular viability and apoptosis were evaluated by CCK-8 assay and flow cytometry, and Cleaved caspase-3 was measured by Immunoblot assay and RT-qPCR. TNF-α, IL-6, and iNOS in CHON-001 cells were determined by RT-qPCR and ELISA. To further verify the effect of TAN IIA on OA, a rat model of OA *in vivo* was established by right anterior cruciate ligament transection. TAN IIA was administered at 50 mg/kg or 150 mg/kg for 7 weeks. The degree of cartilage destruction in OA rats was observed by TUNEL and HE staining. Cleaved caspase-3 and FBXO11 were measured by immunohistochemical staining, RT-qPCR, and Immunoblot. TNF-α, IL-6, and iNOS in chondrocytes of OA rats were detected by ELISA.

**Results:**

IL-1β stimulated CHON-001 cell apoptosis and inflammation, and TAN IIA had anti-apoptosis and anti-inflammatory effects on IL-1β-regulated CHON-001 cells. TAN IIA down-regulated FBXO11 and inhibited PI3K/AKT and NF-κB pathways, thereby alleviating apoptotic and inflammatory reactions in CHON-001 cells under IL-1β treatment. Moreover, TAN IIA treatment improved chondrocyte apoptosis and inflammations in OA rats.

**Conclusion:**

TAN IIA inhibits PI3K/Akt and NF-κB pathways by down-regulating FBXO11 expression, alleviates chondrocyte apoptosis and inflammation, and delays the progression of OA.

## Highlights

• TAN IIA inhibits IL-1β from inducing apoptosis and inflammation in CHON-001 cells.
• FBXO11 overexpression inhibits the protective effect of TAN IIA on apoptosis and inflammation of CHON-001 cells.
• Suppressing PI3K/AKT and NF-κB pathways protects against apoptosis and inflammation of CHON-001 cells.
• TAN IIA treatment improves apoptosis and inflammation of chondrocytes in OA rats.

## Introduction

As an aging process, Osteoarthritis (OA) is characterized by the breakdown of articular cartilage, and the formation of subchondral osteosclerosis and osteophyte.^1^^,^^2^ With high incidences in patients,^3^ OA causes poor quality of life, severe symptoms, and unsatisfactory outcomes. OA involves continuous chondrocyte inflammation and apoptosis,^4^ however, its pathogenesis has not been fully elucidated.

Tanshinone IIA (Tan IIA; C19H18O3, 14, 16-epoxy-20-NOR-5(10),6,8,13, 15-Abietapentaene-11, 12‑dione) is a fat-soluble diterpenoid quinone isolated from Salvia miltiorrhiza.^5^ Salvia miltiorrhiza is widely distributed in China. Aside from promoting blood circulation and removing blood stasis, it alleviates menstrual pain, clears the heart and reduces irritability, and cools blood to alleviate pain. In 1934, Nakao isolated TAN IIA from Salvia miltiorrhiza and identified it as a representative monomer compound.^6^ Because Tan IIA is a quinone-type compound, it has more active electronic properties, is susceptible to REDOX reactions, and can participate in a variety of biochemical reactions.^7^ Several recent studies have shown that TAN IIA can play an anti-apoptotic and anti-inflammatory role. For example, TAN IIA protects acute ethanol-induced myocardial injury *in vivo* and cardiomyocyte apoptosis *in vitro*,^8^ and Tan IIA regulates the expression of the miR-133a-3p/EGFR axis, thereby inhibiting the apoptosis of H9c2 cells *in vivo*.^9^ In addition, Tan IIA plays an anti-inflammatory role by inhibiting iNOS and inflammatory cytokines in RAW 264.7 cells.^10^ Tan IIA can attenuate arthritis, confirming that TAN IIA can also play an anti-inflammatory role *in vivo*.^11^ Although TAN IIA has been reported to inhibit articular cartilage degradation^5^ and alleviate IL-1β-induced chondrocyte inflammatory damage, and is a promising new candidate target for the treatment of arthritis, its anti-apoptotic and anti-inflammatory effects in OA remain unclear and need to be further clarified. At the same time, in clinical application, TAN IIA is mainly used for the treatment of cardiovascular and cerebrovascular diseases,^12^ which has been proven to improve cardiac function. Therefore, TAN IIA has a broad prospect as a clinical drug for the treatment of OA.

FBXO11, a constituent of the F-box protein family, assumes the role of facilitating substrate ubiquitination and degradation, as well as maintaining genome stability. Moreover, FBXO11 functions as a regulator of the TGF-β pathway.^13^ The Jeff mutation is localized within the FBXO11 gene and impairs FBXO11′s capacity to stabilize p53, consequently resulting in the downregulation of the TGF-β/Smad2 pathway. This pathway holds significant importance in the context of immunity and inflammation, as evidenced by similar observations in Fbxo11 knockout mice.^14^ In addition, downregulating FBXO11 in 16HBE cells can reduce apoptosis and inflammation regulated by cigarette smoke extract *in vivo*.^15^ However, the effect of FBXO11 on OA cell apoptosis and inflammation therapy is still weak.

The PI3K/Akt and NF-κB pathways mediate many cell biological activities, such as cell proliferation, apoptosis, and inflammation. Based on previous *in vivo* and *in vitro* experiments, it has been reported that curcumin attenuates high-glucose-induced cardiomyocyte apoptosis by inhibiting NADPH, and this protective effect is mediated by the PI3K/Akt pathway.^16^ Blocking the phosphorylation of the PI3K/AKT signaling pathway mainly mediates anti-inflammation.^17^ NF-κB has an anti-apoptotic effect. For example, vitamin D promotes the anti-apoptotic effect of TNF-α-induced human myeloid nucleus cells by inhibiting the NF-κB pathway.^18^ Meng et al. also demonstrated the anti-apoptotic effect of NF-κB in IVDD rat models.^19^ The NF-kB signaling pathway is considered a typical pro-inflammatory pathway, and studies have shown that OrA activates the NF-κB signaling pathway to reduce LPS-induced inflammatory response and inhibit the expression of iNOS and COX-2 genes through *in vivo* and *in vitro* experiments.^20^ Most importantly, the importance of NF-κB in OA has been demonstrated through *in vivo* and *in vitro* experiments.^21^^,^^22^ NF-κB is activated during aging and inflammation of OA chondrocytes and is widely involved in the pathophysiological processes of OA.

Under normal circumstances, articular cartilage is maintained by a balance between ECM synthesis and degradation, but inflammatory cytokines, especially IL-1β, disrupt this balance and lead to cartilage degradation.^23^ It has previously been reported that OA progression is reduced by inhibiting IL-1β-regulated ERK activation,^24^ and IL-1β down-regulates type II collagen, thus leading to the degradation of articular cartilage.^25^^,^^26^ PI3K/Akt or NF-κB pathway suppression can alleviate apoptosis and inflammation in OA chondrocytes.

Whether Tan IIA inhibits IL-1β-regulated OA chondrocyte apoptosis by activating FBXO11 protein levels remains unclear. This study aimed to explore whether Tan IIA has a definite protective effect on apoptosis and inflammation of OA chondrocytes by regulating FBXO11 and to explore relevant mechanisms involved in the PI3K/Akt and NF-κB pathways on OA progression.

## Materials and methods

### Cell culture and induction

CHON-001 cells (ATCC, USA) were routinely cultured in DMEM plus 10 % FBS (10,099,141, Thermo Fisher Scientific, USA) and 1 U/mL penicillin/streptomycin (10,378,016, Thermo Fisher Scientific). Cells in passages 5‒10 were selected.

For IL-1β treatment, IL-1β (0, 1, 5, and 10 μg/mL) was added to the medium, and finally 10 μg/mL was selected for subsequent experiments. The medium was changed at 70 %‒80 % cell confluence, and cells were divided into different treatment groups. TAN IIA (1,643,339, Sigma, USA) was dissolved in DMSO (Beyotime) to 250 mM and then diluted to 5, 10, 50, and 100 μM. Celecoxib (10 μm, Sigma Aldrich), as a positive agent, was used to treat IL-1β-induced CHON-001 cells.

### Cell transfection and treatment

Transfection was achieved in CHON-001 cells by Lipofectamine 3000 (Thermo Fisher Scientific). pcDNA3.1-FBXO11 was transfected into CHON-001 cells, with pcDNA3.1 (GenePharma, Shanghai, China) as a negative control. Transfection efficiency was evaluated by RT-qPCR.

To explore the effects of inhibition of PI3K/AKT and NF-κB pathways on apoptosis and inflammation of OA chondrocytes, 5 μM PI3K/AKT inhibitor LY294002 (MedChemExpress) or NF-κB inhibitor (PDTC, Sigma) was added to TAN IIA-treated IL-1β-regulated CHON-001 cells.

### Cell viability assay

CHON-001 cells (5 × 10^4^ cells/well) were maintained in 96-well plates of 100 μL medium and attached to the wall after overnight culture. Cells were incubated with 10 μL CCK-8 (Beyotime) for 2 h at 37 °C and analyzed on a Thermomax (Bio-Tek, USA) to read optical density values at 450 nm.

### Flow cytometry

Apoptotic cells were detected by Annexin V-FITC apoptosis detection kit (Thermo Fisher Scientific). CHON-001 cells were put in 6-well plates with 5 × 10^4^ cells/well overnight, digested with EDTA-free trypsin (0.25 %, Beyotime), and centrifuged at 1200 rpm for 5 min. The cell precipitations were suspended in 500 μL 1 × Annexin V binding buffer and stained with 10 μL Annexin V-FITC and 5 μL PI for 30 min away from light. Apoptotic cells (Annexin *V*^+^ and PI^−^) were distinguished from necrotic cells (Annexin *V*^+^ and PI^+^) on the FACS Calibur flow cytometry (BD Biosciences, USA).

### TUNEL assay

Cartilage tissues were fixed with 4 % paraformaldehyde (Beyotime) for 30 min, permeated with 0.1 % Triton-X 100 for 3 min, stained with *in situ* apoptosis detection kit (Sigma-Aldrich), and then re-stained with DAPI for 10 min. Images were taken under a confocal microscope (Nikon Eclipse 80i, Japan).

### RT-qPCR assay

Total RNA was isolated from CHON-001 cells and tissues using TRIzol reagent (Invitrogen, USA) and processed with Prime Script RT Reagent Kit (Takara). The obtained cDNA was considered a template for SYBR Green qRT-PCR (Takara) analysis. PCR was implemented with a 7500 real-time fluorescent quantitative PCR system (Applied Biosystems, USA) and SYBR Premix Ex Taq Kit (Takara), and mRNA expression was standardized at GAPDH levels. Specific primers designed for target mRNA are shown in Table 1. The results are shown as relative expressions, and the formula is the 2^−ΔΔCT^ method.

**Table 1:** Primer Sequence.

**Gene**	**Forward primer (5′→3′)**	**Reverse primer (5′→3′)**
IL-1β	5′-GCCCATCCTCTGTGACTCAT-3′	5′-GAAGGTCCACGGGAAAGACAC-3′
IL-6	5′-CCTGAACCTTCCAAAGATGGC-3′	5′-TTCACCAGGCAAGTCTCCTCA-3′
iNOS	5′-CTGCAGCACTTGGATCAG GAACCTG-3′	5′-GGGAGTAGCCTG TGTGCACCTGGAA-3′
TNF-α	5′-ACGGCATGGATC TCAAAGAC-3′	5′-GTGGGTGAGGAGCACGTAGT-3′
FBXO11	5ʹ-GGTCATCGTGCAAAACGTGC-3ʹ	5ʹ-ACAAGCTGCTCTACAAAGATCC-3ʹ
Cleaved caspase-3	5ʹ-CTCTGGACTGCTGCATGGTG-3ʹ	5ʹ-TCTCTCGACGGACACAGGAC-3ʹ
GAPDH	5′-CATCATCCCTGCCTCTACTGG-3′	5′-GTGGGTGTCGCTGTGTGAAGTC-3′

### Immunoblot assay

The collected cells and tissues were lysed in a RIPA lysis buffer mixed with protease inhibitor and phosphatase inhibitor (Roche, Switzerland) for 30 min. After detection with the BCA Protein Assay Kit (Beyotime), proteins were isolated on 10 % SDS-PAGE, loaded to a PVDF membrane (Millipore, USA), and sealed with 5 % skim milk for 2 h. Specific primary antibodies β-actin (15,204–1-p, Proteintech), Cleaved caspase-3 (Asp175, Cell Signaling Technology, USA), FBXO11 (ab181801, Abcam), iNOS (ab178945, Abcam), PI3K (ab302958, Abcam), p-PI3K, AKT (ab8805, Abcam), p-AKT, p65 (ab111577, Abcam), and p-p65 were incubated overnight and washed in three-buffered saline containing 0.1 % Tween-20. The secondary antibody coupled with horseradish peroxidase was reacted for 6 h. Protein bands were developed by the Chemo Dox XRS system (Bio-Rad, USA), of which the optical density was calculated by ImageJ version 6.0.

### ELISA test

As requested by the manufacturer, TNF-α, IL-6, and iNOS in tissues and cells were determined using ELISA Kits (R&D Systems, USA).

### Rat oa model

All procedures were conducted in accordance with the ARRIVE Guidelines, and the Animal Research Ethics Committee of Shanghai Baoshan Hospital of Integrated Traditional Chinese and Western Medicine (n° 2020B0633) approved all rat experiments. The Experimental Animal Center of Shanghai Baoshan Hospital of Integrated Traditional Chinese and Western Medicine provided 50 male Sprague Dawley rats (10‒12 weeks, 250‒300 g). An Anterior Cruciate Ligament Transaction (ACLT) at the right knee induced OA. Under anesthesia with an intraperitoneal injection of 50 mg/L chloral hydrate (Beyotime), the rats were placed with the left posterior region fixed in a supine position. The medial joint capsule was incised, the extensor muscles were gently displaced laterally without severing the patellar ligament, the anterior cruciate ligament was severed, and the joint was rinsed with sterile saline. The joint was then closed with 7‒0 surgical sutures and the skin wound was closed. To prevent wound infection, amoxicillin was applied topically, and rat status was monitored.^30^ Another 10 rats that did not undergo ACLT surgery served as a control group. The rats were randomly divided into 5 groups: sham group, ACLT group, ACLT + celecoxib (10 mg/kg) group, ACLT + low-dose TAN IIA group, and ACLT + high-dose TAN IIA group. After one week of adaptive feeding, celecoxib was dissolved in normal saline containing 0.1 % DMSO and administered orally at a dose of 10 mg/kg/day. TAN IIA was injected once a day into the peritoneum of the rats at either a low dose (50 mg/kg) or a high dose (150 mg/kg) for 7-weeks. After euthanized rats, knee joint samples were collected for follow-up analysis.

### HE-staining

Cartilages were fixed in 4 % paraformaldehyde for 24 h and immersed in 10 % EDTA for 2‒3 weeks. After dehydration, the cartilages were embedded in paraffin and sectioned to 5 μm. HE-staining was conducted (Beyotime) and samples were observed under a microscope (Leica).

### Immunohistochemistry (IHC)

Cartilages were fixed in 4 % paraformaldehyde and prepared into paraffin slices with 5 mm thickness. The slices were dewaxed and treated with 3 % H_2_O_2_ for 15 min and sealed with 5 % normal serum for 30 min. After Cleaved caspase-3 and FBXO11 antibody treatment at 4 °C, the slices were incubated with the secondary antibody and imaged under an optical microscope. Images were analyzed by ImageJ 6.0.

### Data analysis

SPSS20 statistical software analyzed the experimental data. Measurement data were expressed as mean ± SD and conditioned to a comparative analysis by *t*-test or one-way ANOVA, with * *p* < 0.05 emphasizing statistical significance. All analyses were performed using GraphPad Prism 8.0.

## Results

### TAN iia inhibits IL-1β from inducing apoptosis and inflammation in CHON-001 cells

**Fig. 1:**
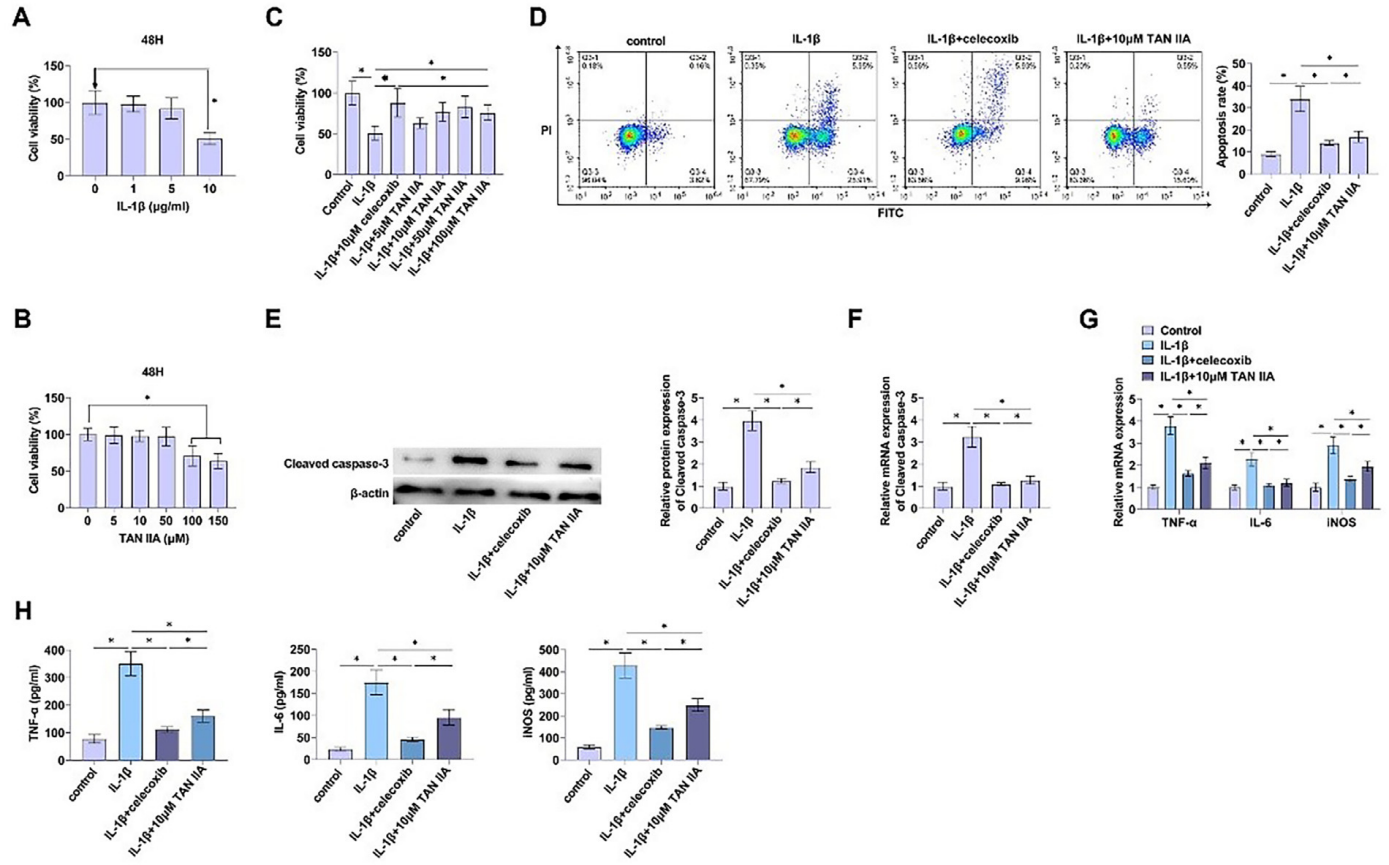
TAN IIA inhibits IL-1β from inducing apoptosis and inflammation in CHON-001 cells. (A) Cytotoxicity of IL-1β on CHON-001 cells. (B) Cytotoxicity of TAN IIA on CHON-001 cells. (C) Viability of CHON-001 cells stimulated by IL-1β (10 μg/mL) treated with TAN IIA at different concentrations. (D) Apoptosis of CHON-001 cells after Annexin V-FITC and PI staining. (E‒F) Immunoblot and RT-qPCR detection of Cleaved caspase-3. (G‒H) TNF-α, IL-6, and iNOS determined by RT-qPCR and ELISA. Data are expressed as mean ± SD (*n* = 3) (* *p* < 0.01).

Based on CCK-8, the cell viability of CHON-001 showed a significant decrease at an IL-1β concentration of 10 μg/mL (Fig. 1A). Therefore, CHON-001 cells were treated with 10 μg/mL IL-1β to establish a cell model.

The effect of different concentrations of TAN IIA on IL-1β-regulated CHON-001 cell viability was determined by CCK-8 assay, and it was found that under IL-1β treatment, there was no cytotoxicity when TAN IIA concentration was less than 100 μM, and 10‒100 μM was the most suitable dose range for studying the effect of TAN IIA (Fig. 1B). IL-1β-induced CHON-001 cells were treated with 0‒100 μM TAN IIA for 48 h, and celecoxib significantly rescued IL-1β-induced cytotoxicity of CHON-001 cells, while 10‒100 μM TAN IIA significantly reduced IL-1β-induced cytotoxicity in a dose-dependent manner (Fig. 1C). Therefore, to mimic the OA treatment model, CHON-001 cells were exposed to IL-1β at 10 μg/mL for 48 h, followed by treatment with TAN IIA at 10 μM for the same duration.

The anti-apoptotic and anti-inflammatory effects of TAN IIA in CHON-001 chondrocytes were analyzed *in vitro*. Flow cytometry evaluated that apoptosis was significantly increased after IL-1β treatment, while TAN IIA or celecoxib significantly inhibited the induction of apoptosis by IL-1β and reversed the promoting effect of IL-1β on apoptosis of CHON-001 cells (Fig. 1D). The results were further confirmed by Western Blot and RT-qPCR detection of apoptosis-related proteins. TAN IIA or celecoxib inhibited expression of the pro-apoptotic protein Cleaved caspase-3 (Fig. 1E, 1F).

TNF-α, IL-6, and iNOS in CHON-001 cells were measured by RT-qPCR, and the three indices were upregulated by IL-1β, but TAN IIA or celecoxib TAN IIA or celecoxib significantly reversed this effect, inhibiting the production of pro-inflammatory factors (Fig. 1G). ELISA results were consistent with RT-qPCR results (Fig. 1H).

### TAN iia regulates FBXO11 expression

**Fig. 2:**
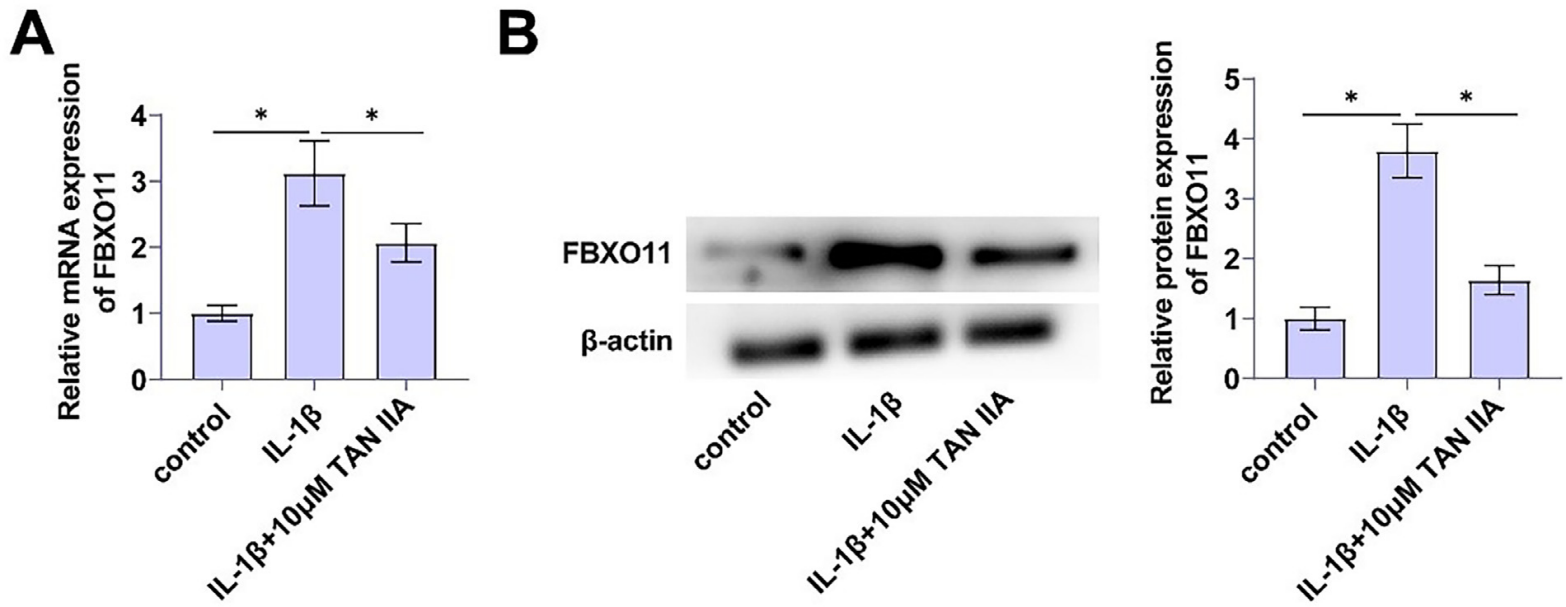
TAN IIA regulates FBXO11 expression. (A‒B) FBXO11 in designated treated CHON-001 cells measured by RT-qPCR and Immunoblot assay. Data are expressed as mean ± SD (*n* = 3) (* *p* < 0.05).

To determine the regulation of FBXO11 by TAN IIA and its role in CHON-001 cells, FBXO11 in CHON-001 cells was measured by RT-qPCR. IL-1β promoted FBXO11 expression, while TAN IIA could eliminate the pro-inflammatory effects of IL-1β on FBXO11 expression (Fig. 2A). Immunoblot results showed the same trend (Fig. 2B).

### FBXO11 overexpression inhibits the protective effect of tan iia on apoptosis and inflammation of CHON-001 cells

**Fig. 3:**
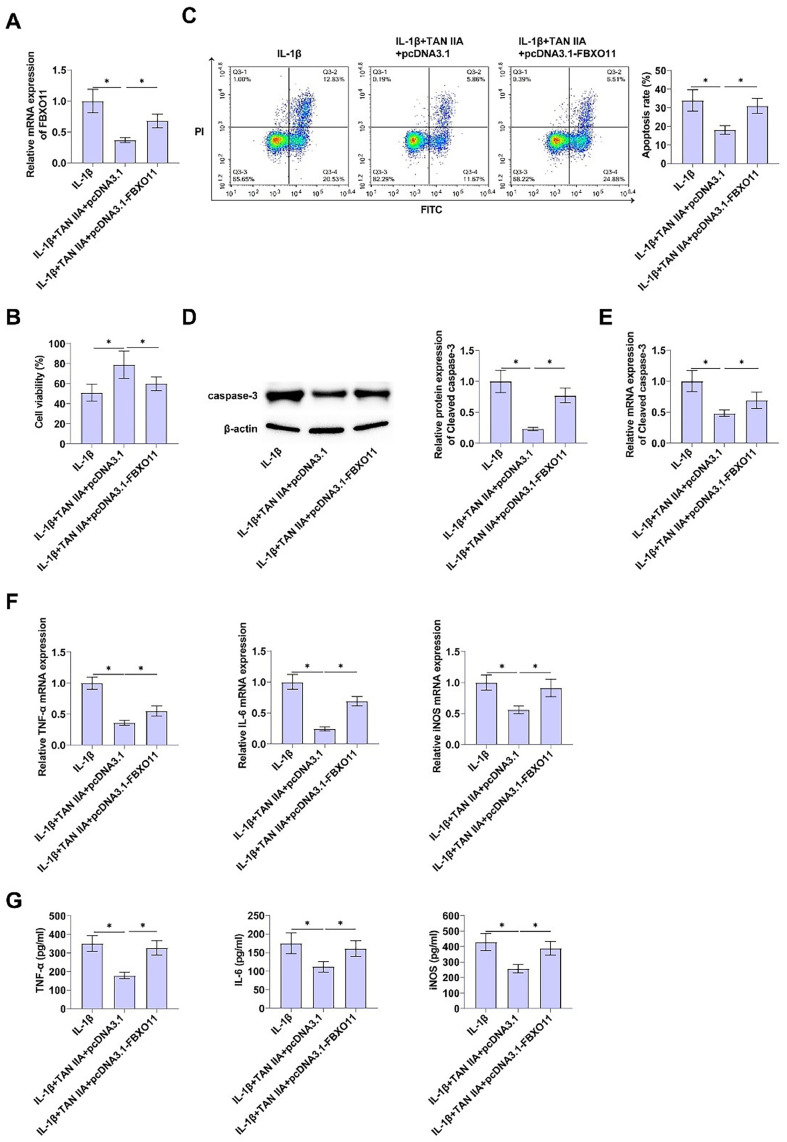
Elevating FBXO11 inhibits the protective effect of TAN IIA on apoptosis and inflammation of CHON-001 cells pcDNA3.1-FBXO11 was transfected into TAN IIA-treated CHON-001 cells. (A) FBXO11 in designated treated CHON-001 cells measured by RT-qPCR. (B) CCK-8 assay evaluated proliferation of CHON-001 cells. (C) Apoptosis of CHON-001 cells after Annexin V-FITC and PI staining. (D‒E) Immunoblot and RT-qPCR detection of Cleaved caspase-3. (F‒G) TNF-α, IL-6, and iNOS determined by RT-qPCR and ELISA. Data are expressed as mean ± SD (*n* = 3) (* *p* < 0.01).

To verify whether FBXO11 is involved in TAN IIA's inhibition of apoptosis and inflammation of CHON-001 cells and the relationship between them, pcDNA3.1-FBXO11 was transfected into CHON-001 cells treated with TAN IIA. RT-qPCR determined that after transfection for 24 h, FBXO11 mRNA in the pcDNA3.1 group and the control group had no significant change, while increased in the PCDNA3.1-FBXO11 group (Fig. 3A), indicating successful transfection.

CCK-8 evaluated the effect of TAN IIA on CHON-001 cell viability after transfection with pcDNA3.1-FBXO11, and upregulation of FBXO11 significantly inhibited the promotion of TAN IIA on CHON-001 cell viability (Fig. 3B). Flow cytometry results showed that overexpressing FBXO11 weakened the anti-apoptotic effect of TAN IIA on CHON-001 cells (Fig. 3C). Immunoblot and RT-qPCR assay showed that pcDNA3.1-FBXO11 increased Cleaved caspase-3 levels (Fig. 3D, 3E), promoting apoptosis of CHON-001 cells.

RT-qPCR detected that overexpression of FBXO11 significantly increased TNF-α, IL-6, and iNOS mRNA, and inhibited the anti-inflammatory effect of TAN IIA on CHON-001 cells (Fig. 3F). In addition, ELISA also found consistent results, indicating that upregulation of FBXO11 promoted TNF-α, IL-6, and iNOS contents (Fig. 3G).

### FBXO11 activates PI3K/AKT and nf-κb pathways

**Fig. 4:**
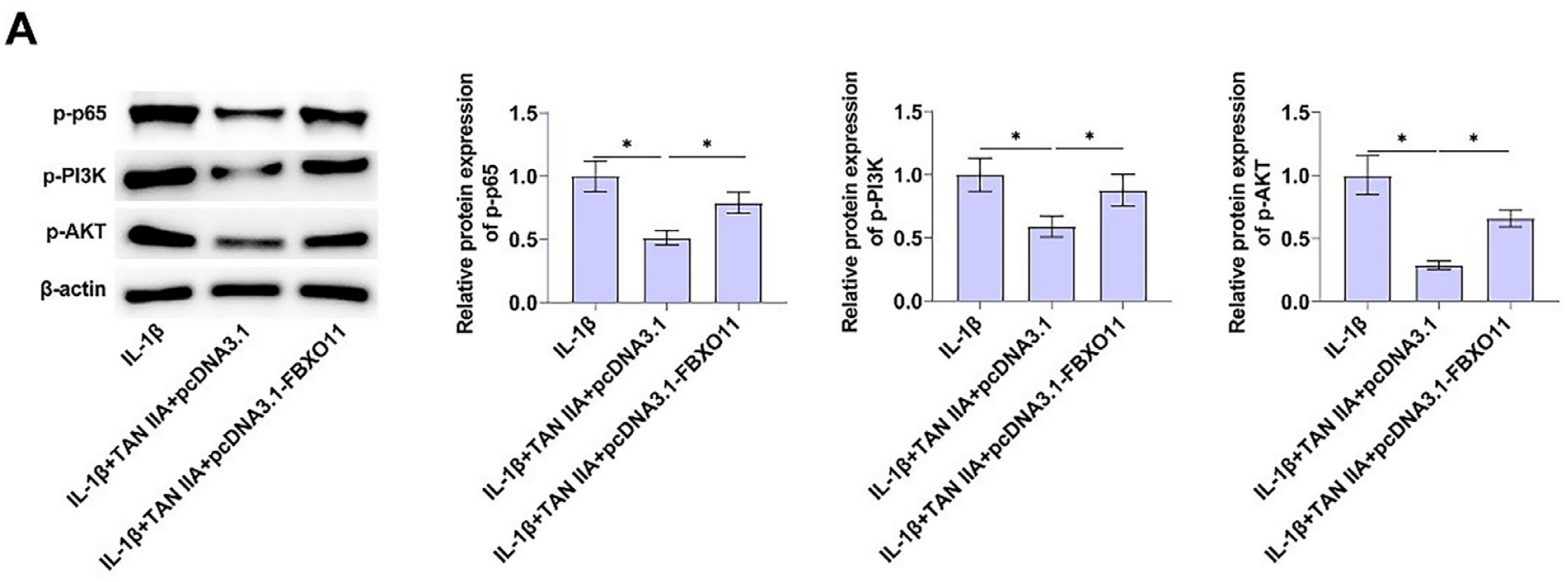
FBXO11 activates PI3K/AKT and NF-κB pathways. Immunoblot determined related proteins in PI3K/AKT and NF-κB pathways after pcDNA3.1-FBXO11 transfection. Data are expressed as mean ± SD (*n* = 3) (* *p* < 0.05).

According to the present results, TAN IIA inhibits IL-1β-induced apoptosis of CHON-001 chondrocytes by down-regulating FBXO11 expression. To further investigate the relationship between FBXO11 and PI3K/AKT and NF-κB pathways, immunoblot analysis was performed to detect the effect of transfection of pcDNA3.1-FBXO11 on the expression level of related proteins in PI3K/AKT and NF-κB pathways. FBXO11 overexpression upregulated p-PI3K, p-AKT, and p-p65 in CHON-001 cells (Fig. 4).

### Suppressing PI3K/AKT and nf-κb pathways protects against apoptosis and inflammation of CHON-001 cells

To investigate the potential involvement of the PI3K/AKT and NF-κB pathways in the effects of TAN IIA on apoptosis and inflammation in CHON-001 cells, subsequent to TAN IIA pretreatment, the PI3K/AKT pathway inhibitor LY294002 or the NF-κB pathway inhibitor PDTC was introduced to the CHON-001 cells.

**Fig. 5:**
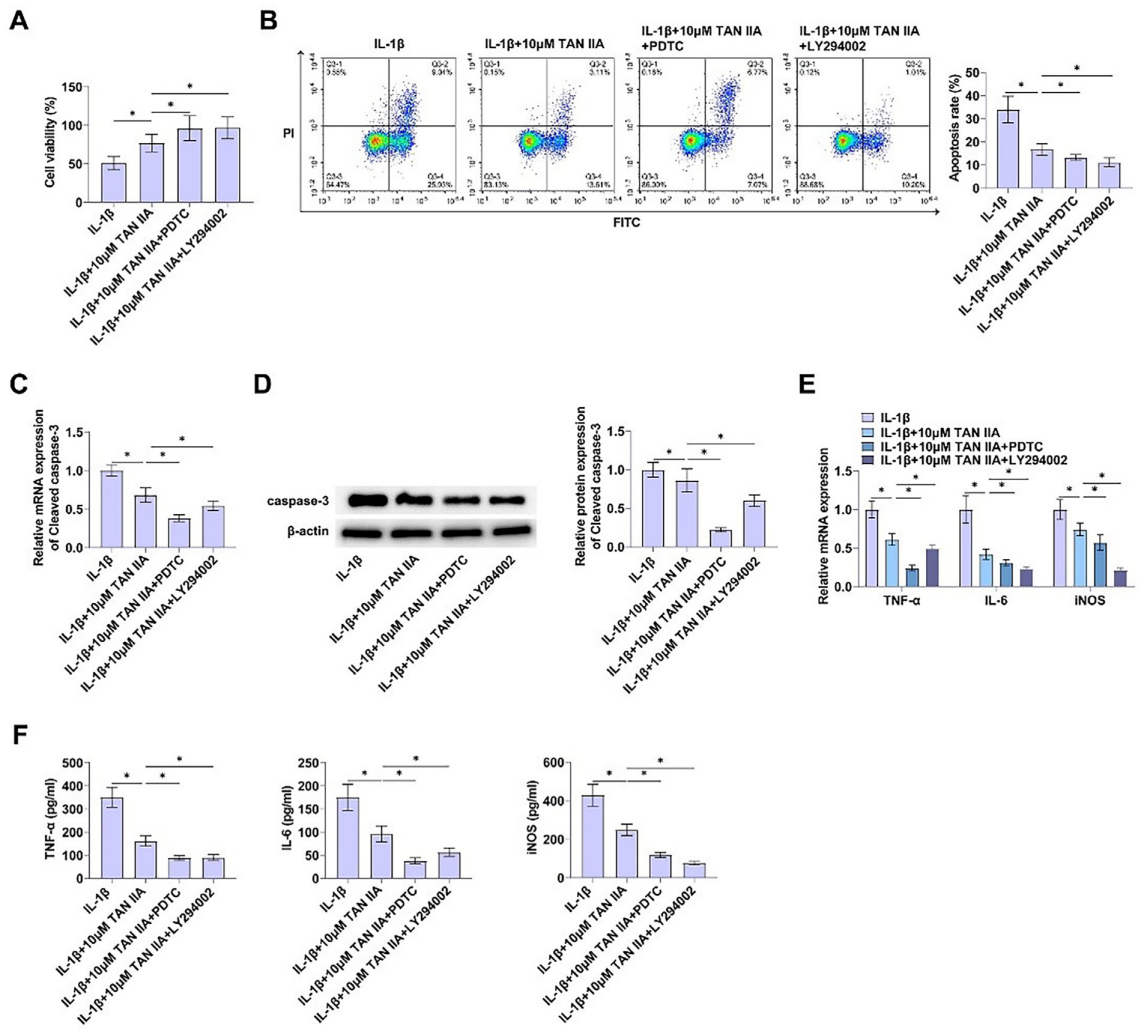
Inhibition of PI3K/AKT and NF-κB pathways partially reverses IL-1β-regulated apoptosis and inflammation in CHON-001 cells. (A) CCK-8 assay evaluated proliferation of CHON-001 cells. (B) Apoptosis of CHON-001 cells after Annexin V-FITC and PI staining. (C‒D) Immunoblot and RT-qPCR detection of Cleaved caspase-3. (E‒F) TNF-α, IL-6, and iNOS determined by RT-qPCR and ELISA. Data are expressed as mean ± SD (*n* = 3) (*p < 0.05).

The effect of PI3K/AKT and NF-κB signaling pathway inhibitors on TAN IIA's promotion of CHON-001 cell viability was investigated by CCK-8 assay, and the results showed that LY294002 and PDTC promoted the protective effect of TAN IIA on CHON-001 cell apoptosis (Fig. 5A). Flow cytometry found that LY294002 and PDTC inhibited CHON-001 cell apoptosis and promoted the anti-apoptotic effect of TAN IIA (Fig. 5B). In addition, RT-qPCR and immunoblot analysis showed that LY294002 and PDTC reduced Cleaved caspase-3 expressions (Fig. 5C, 5D).

RT-qPCR showed that after treatment with LY294002 or PDTC, TNF-α, IL-6 and iNOS mRNA was decreased, which promoted the anti-inflammatory effect of TAN IIA on IL-1β-induced CHON-001 cells (Fig. 5E). In addition, ELISA assay showed that TNF-α, IL-6 and iNOS contents were inhibited by LY294002 or PDTC treatment (Fig. 5F).

### TAN iia treatment improves apoptosis and inflammation of chondrocytes in oa rats

TAN IIA in OA was further studied by establishing a rat model of OA *in vivo*. The OA model was established by surgical transection of the anterior cruciate ligament of the right knee, and the rats were injected intraperitoneally once a day with either 50 mg/kg or 150 mg/kg TAN IIA. The rats were euthanized after 7-weeks, and tissue samples were collected.

**Fig. 6:**
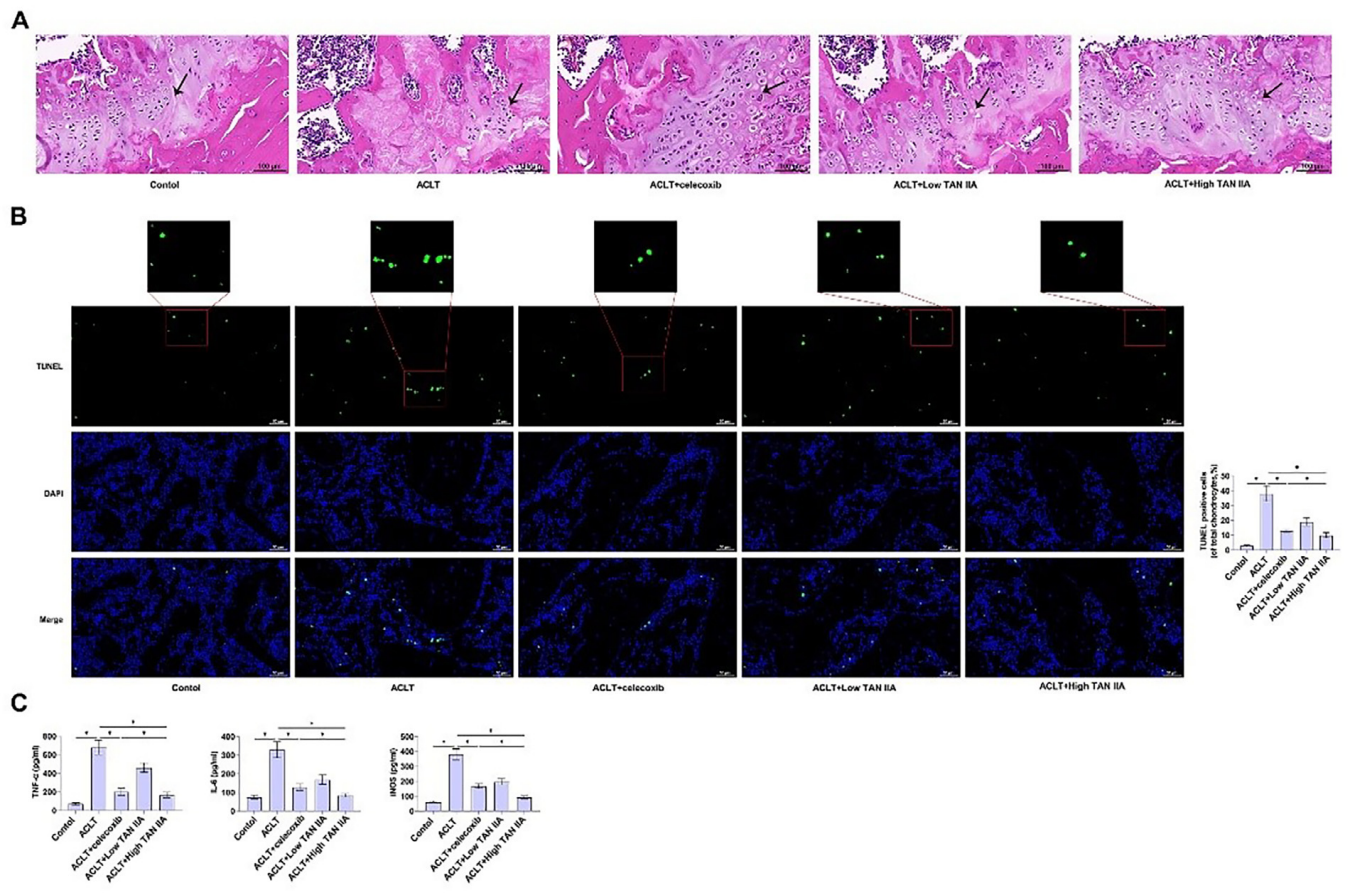
TAN IIA treatment improves apoptosis and inflammation of chondrocytes in OA rats. (A) HE-staining of articular cartilage. (B) TUNEL staining determined apoptosis of rat cartilage. (C) TNF-α, IL-6, and iNOS in chondrocytes of OA rats detected by ELISA. Data are expressed as mean ± SD (*n* = 10). (*p < 0.01).

To evaluate the effect of TAN IIA on cartilage degeneration and osteophyte formation in ACLT rats, HE-staining was used for histological analysis. HE-staining showed that articular cartilage was damaged and destroyed, and chondrocytes were reduced in OA rats. TAN IIA or celecoxib improved cartilage injury and delayed OA progression in OA rats (Fig. 6A). TUNEL staining measured apoptosis in rat cartilage tissue samples, and the percentage of apoptosis was significantly higher in OA rats, while TAN IIA reduced apoptosis levels in a dose-dependent manner (Fig. 6B). To verify the mechanism by which TAN IIA inhibited chondrocyte apoptosis in the treatment of OA by regulating FBXO11, expression levels of Cleaved caspase-3, were measured. IHC staining determined that TAN IIA reduced Cleaved caspase-3 and FBXO11 intensity in the OA cartilage (Supplementary Fig. 1A). RT-qPCR and immunoblot assay were consistent with IHC results, and these markers were significantly increased after ACLT surgery, and TAN IIA significantly decreased Cleaved caspase-3 and FBXO11 expression (Supplementary Fig. 1B, 1C).

To verify the effect of TAN IIA treatment on OA synovial inflammation, the effect of TAN IIA on OA rat chondrocyte inflammation was evaluated by ELISA experiment, and the levels of TNF-α, IL-6, and iNOS in OA rat tissues were detected. After TAN IIA treatment, TNF-α, IL-6, and iNOS in OA rat chondrocytes were significantly decreased, indicating that TAN IIA had an anti-inflammatory effect on OA rat chondrocytes (Fig. 6C).

## Discussion

OA progression is attributed to apoptosis and inflammation of a large number of chondrocytes, which leads to the degeneration of cartilage and thickening of subchondral bone.^22^ Current OA treatment strategies to relieve pain symptoms are limited, and although medications can be used to relieve pain, serious side effects often occur. TAN IIA is a phytochemical that has anti-apoptotic, anti-inflammatory bioactivity, and research on its role in OA therapy remains limited. This study demonstrated that TAN IIA inhibits PI3K/Akt and NF-κB pathways by regulating FBXO11 expression, thereby alleviating apoptosis and inflammation of OA chondrocytes.

The induction of OA is attributed to the release of inflammatory cytokines, with IL-1β playing a significant role in disrupting the typical structure and function of chondrocytes. This disruption leads to chondrocyte apoptosis, degradation of chondrocyte Extracellular Matrix (ECM), involvement in synovial inflammatory lesions, and impact on bone metabolism. Moreover, the ACLT rat model is widely recognized as the standard OA model and has been extensively employed in numerous research studies.^27^ Therefore, in this study, IL-1β was used to induce CHON-001 cells to establish an inflammatory environment *in vitro*. ACLT rat models were used to simulate OA progression *in vivo*.

There are several therapeutic effects of Tan IIA, a compound isolated from Salvia miltiorrhiza, including pro-apoptotic and anti-inflammatory actions. TAN IIA alleviates inflammation, oxidative stress, and apoptosis induced by mouse protocells by inhibiting the PI3K/Akt/FoxO1 pathway.^28^ and TAN IIA inhibits apoptosis in cirrhotic rat models by activating Akt and inhibiting p38 MAPK.^29^ Therefore, Tan IIA is feasible in OA therapeutic applications. To test this hypothesis, IL-1β-stimulated chondrocytes were treated with TAN IIA. TAN IIA promoted chondrocyte proliferation and viability in a concentration-dependent manner at 0‒50 μM but was inhibited by TAN IIA at 100‒150 μM. This was demonstrated by histomorphologic analysis of the TAN IIA group compared with the control group and by immunohistochemical analysis of Cleaved Caspase-3 expression. In a previous study,^2^ after treating chondrocytes with 0‒50 μM TAN IIA for 72 h, 0‒20 μM TAM IIA showed no toxicity, and when the concentration of Tan IIA exceeded 40 μM, it produced cytotoxicity to chondrocytes. In this study, 0‒150 μM TAN IIA was treated for 48 h, and the results showed that there was no cytotoxicity when TAN IIA concentration was less than 100 μM, and 100‒150 μM TAN IIA significantly reduced the viability of chondrocytes. Therefore, it is speculated that the effect of TAN IIA on chondrocytes is closely related to the concentration level, and further studies are still needed to clarify. Importantly, 10 or 20 μM TAN IIA inhibited 20 ng/mL TNF-α-induced apoptosis and inflammatory cytokine production. In addition, 1 μM TAN IIA derivative (sodium TAN IIA sulfonate, STS) can reduce apoptosis and inflammation of chondrocytes.^30^ These studies show that TAN IIA has proven anti-apoptotic and anti-inflammatory effects despite the different concentrations of action of TAN IIA and its derivatives.

To further emphasize the potential of Tan IIA as a treatment for OA, the therapeutic effect of this compound was verified using ACLT rat models. The ACLT model has been extensively employed to elucidate OA pathogenesis and investigate potential therapeutic targets, including the validation of the therapeutic efficacy of novel medications.^3^ Due to the poor solubility of Tan IIA, studies have shown that they exhibit a strong first-pass effect after oral administration and are excreted through the liver.^31^ In addition, Tan IIA is easily eliminated from the bloodstream after intravenous injection, so it has a short shelf life. STS is the most commonly used of the drugs currently available. In experimental studies, STS and TAN IIA were utilized interchangeably; however, their distinct chemical structures resulted in dissimilar biological effects and pharmacological activities. Consequently, the reliability of interchangeably applying STS and TAN IIA is compromised. The alteration of TAN IIA to STS not only modifies its molecular structure, chemical properties, and pharmacokinetics, but also impacts its pharmacological activity.^31^ In a previous study, peritoneal injection was also used for dosing.^2^ Therefore, TAN IIA was administered to rats by intraperitoneal injection. In addition, celecoxib was used as a positive control drug in this study. Celecoxib was the first approved COX-2-specific inhibitor to significantly improve pain and inflammation in OA and rheumatoid arthritis. Compared with non-steroidal anti-inflammatory drugs, it is more safe to protect the gastrointestinal tract and can continuously relieve OA symptoms.^32^ The present results showed that celecoxib had a significant effect on the improvement of OA and reduced the inflammatory environment. The symptoms of ACLT rats treated with Tan IIA were significantly reduced, the level of inflammatory factors was significantly reduced, and the pathological manifestations were improved. More importantly, low-dose TAN IIA had less anti-apoptotic and anti-inflammatory capacity than celecoxib, while high-dose TAN IIA showed a greater OA improvement advantage than celecoxib. At the same time, the anti-inflammatory effect of TAN IIA on IL-1β-stimulated chondrocytes was verified by *in vitro* experiments. These data all confirm the present study's hypothesis that TAN IIA has the potential to treat OA.

At present, studies on the FBXO11 gene mainly focus on various tumors, such as renal cell carcinoma, gastric cancer, human B-cell lymphoma, etc.^33^, ^34^, ^35^ Other diseases related to FBXO11 gene variation include chronic otitis media, vitiligo, etc. For the mechanism of OA, FBXO11 has not been deeply studied in this respect. In the present study, TAN IIA protected against apoptosis and inflammation in OA by down-regulating FBXO11 expression. FBXO11 was involved in apoptosis and inflammation of NT-AS1/miR-582–5p/FBXO11 pathway induced by CSE,^36^ and FBXO11 could regulate miR-26a to inhibit the proliferation, migration, and invasion of liver cancer cells.^37^ In these experiments, TAN IIA regulated FBXO11 and inhibited its expression in OA chondrocytes, and its knockdown promoted the anti-apoptotic and anti-inflammatory effects of TAN IIA, which was also verified *in vivo*. FBXO11 serves as a substrate recognition component that encodes the SKP1-cullin-F-box complex, responsible for the ubiquitination and subsequent degradation of substrates, thereby contributing to the maintenance of genomic stability. Furthermore, FBXO11 assumes a regulatory role in the cell cycle by facilitating substrate degradation, consequently influencing the apoptosis process.^38^ Simultaneously, FBXO11 serves as a regulator of the TGF-β pathway, and the Jeff mutation is localized within the FBXO11 gene, thereby impacting the capacity of FBXO11 to maintain the stability of p53. Consequently, this disruption leads to an alteration in the TGF-β/Smad2 signaling pathway, thereby signifying the significant involvement of FBXO11 in the inflammatory process.

The impact of Tan IIA on the proliferation, invasion, and migration of tumor cells has been documented through various signaling pathways.^39^ Nevertheless, the precise molecular mechanism by which Tan IIA affects OA remains undisclosed. The PI3K/AKT and NF-κB pathways play a crucial role in chondrocyte apoptosis and inflammation,^22^ and comprehending their regulation would greatly contribute to the management of OA. Activation of the PI3K/AKT pathway seems to be a pivotal factor in promoting proliferation and anti-apoptotic responses, which are characteristic of inflammatory processes in OA tissue. In the present data, Tan IIA was found to block IL-1β-stimulated protein phosphorylation associated with the PI3K/AKT and NF-κB pathways. These results suggest that Tan IIA exerts anti-apoptotic and anti-inflammatory activities through PI3K/AKT and NF-κB pathways.

Combined with previous studies, it is possible to improve clinical symptoms of OA with Tan IIA alone or in combination with other drugs. However, when applied to actual clinical treatment, the oral dose and specific protective effect of TAN IIA still need to be further studied on TAN IIA in clinical practice and the detailed physiological mechanism. Secondly, this study focused on the effects of TAN IIA on chondrocytes and cartilage tissue, excluding the effects on other related cells and tissues.

In brief, this study investigated the effect of TAN IIA on IL-1β-regulated chondrocyte apoptosis and inflammation and its potential pathways and again confirmed that IL-1β stimulated chondrocyte apoptosis and inflammatory response, and demonstrated that TAN IIA regulated FBXO11 expression, inhibited PI3K/Akt and NF-κB pathway, alleviated IL-1β-regulated chondrocyte apoptosis and inflammation. This study helps us further understand the protective effect of TAN IIA and suggests that TAN IIA may be an effective new therapeutic agent to delay OA progression.

## Availability of data and materials

The datasets used and/or analyzed during the present study are available from the corresponding author upon reasonable request.

## Ethics statement

The present study was approved by the Shanghai Baoshan Hospital of Integrated Traditional Chinese and Western Medicine (n° 2020B0633) Animal Experimental Ethics Committee. All procedures complied with the National Institutes of Health Guide for the Use of Laboratory Animals.

## Authors' contributions

Jin Xu designed the research study. Jin Xu and Ren Ding performed the research. Xiao Cheng Zhi and Yun Hui Zhang provided help and advice. Xiao Cheng Zhi and Yun Hui Zhang analyzed the data. Jin Xu wrote the manuscript. Jin Xu and Ren Ding reviewed and edited the manuscript. All authors contributed to editorial changes in the manuscript. All authors read and approved the final manuscript.

## Funding

Excellent Young Medical Talents Training Program of Shanghai Baoshan Hospital of Integrated Chinese and Western Medicine (n° 2021BY001)

## Declaration of competing interest

The authors declare no conflicts of interest.

## Appendix Supplementary materials

Image, application 1
