# Supplementary material for: Tanshinone IIA alleviates IL-1β-induced chondrocyte apoptosis and inflammation by regulating FBXO11 expression
Source: Clinics (Sao Paulo). 2024 Apr 26;79:100365. doi: 10.1016/j.clinsp.2024.100365 (PMC11061256; doi:10.1016/j.clinsp.2024.100365)

CLINICS-D-23-00798_Supplementary Material

**Supplementary Figure 1** Expression levels of Cleaved caspase-3 and FBXO11 in OA rats. (A) Cleaved caspase-3 and FBXO11 measured by IHC. (B‒C) Cleaved caspase-3 and FBXO11 detected by RT-qPCR and immunoblot. Data are expressed as mean ± SD (n = 10) (* p < 0.01).


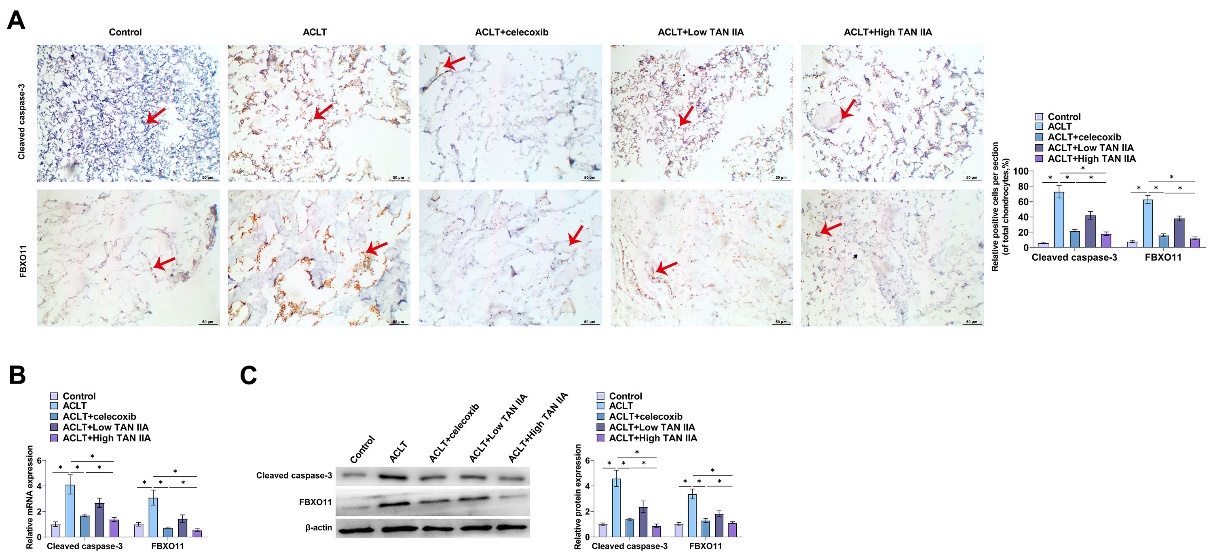

Supplement: Supplementary file 1 [file mmc1.docx]
